# Effectiveness of Baby Friendly Community Initiative (BFCI) on complementary feeding in Koibatek, Kenya: a randomized control study

**DOI:** 10.1186/s12889-018-5519-1

**Published:** 2018-05-08

**Authors:** Mildred Maingi, Judith Kimiywe, Sharon Iron-Segev

**Affiliations:** 1 School of Nutritional Sciences, The Hebrew University of Jerusalem and The International School of Agricultural Sciences, P.O Box 12-761001, Rehovot, Israel; 2 Department of Food Nutrition and Dietetics, Kenyatta University, P.O Box 43844-00100, Nairobi, Kenya

**Keywords:** Baby Friendly Community Initiative, Complementary feeding

## Abstract

**Background:**

Appropriate infant and young child nutrition is critical for proper growth and development. In order to promote optimal nutrition at an early age, the World Health Organization (WHO) and UNICEF have developed the Baby Friendly Hospital Initiative (BFHI) to address poor breastfeeding practices in maternity wards. However, impact is limited in less developed countries like Kenya, where more than half of all births are home deliveries. Therefore, Kenya has explored the adoption of Baby Friendly Community Initiative (BFCI) in its rural settings. In contrast to the BFHI, the BFCI supports breastfeeding and optimal infant feeding in community. BFCI has been implemented in Koibatek, in rural Kenya. This study aimed at assessing the effectiveness of BFCI on complementary feeding practices of children aged 6–23 months, by comparing intervention and control groups.

**Methods:**

This was a randomized control study design that included 270 mother-infant pairs enrolled in the Baby Friendly Community Initiative (BFCI) project in Koibatek. Evaluation was carried out using structured questionnaires.

**Results:**

A statistically significantly higher proportion of children in the intervention group compared to the control group attained minimum dietary diversity (77% vs. 58%; *p* = 0.001), minimum meal frequency (96% vs. 89%; *p* = 0.046) and minimum acceptable diet (77% vs. 61%; *p* = 0.005). The odds of attaining minimum dietary diversity, minimum meal frequency and minimum acceptable diet were statistically significantly higher for the intervention group compared to control group (OR: 4.95; 95%CI 2.44–10.03, *p* = < 0.001; OR: 14.84; 95%CI 2.75–79.9, *p* = 0.002; OR: 4.61; 95%CI 2.17–9.78, *p* = < 0.001 respectively).

**Conclusion:**

The BFCI intervention was successful in improving complementary feeding practices. Strengthening and prioritizing BFCI interventions could have a significant impact on child health outcomes in rural Kenya.

**Trial registration:**

ISRCTN03467700. Registration 24 September 2014. Retrospectively registered.

## Background

Appropriate infant and young child nutrition (IYCN) is critical for optimal growth and development [1]. Poor nutrition in childhood is associated with negative outcomes on cognitive development, morbidity in later life and overall economic productivity [2, 3]. The World Health Organization (WHO) recommends exclusive breastfeeding of infants for the first 6 months of life, with continued breastfeeding together with proper complementary feeding for up to 2 years of age [4]. In Kenya, poor IYCN is still a major challenge. According to the Kenya Demographic Health Survey (KDHS) 2014, stunting levels were 26%, while severe stunting was observed in 12% of children aged 18 to 23 months. Additionally, data from KDHS 2014 indicated that only 22% of children in Kenya were fed according to the WHO recommended feeding practices [5]. Rural settings of Kenya have high rates of acute malnutrition, which is primarily attributed to poor child feeding practices, poor health-seeking behavior and poor sanitation and hygiene [6].

In order to address poor IYCN practices, improve health and reduce mortality in Kenya, the child survival and development strategy aims at accelerating and scale up of evidence-based high impact interventions [7]. The Ministry of Health in Kenya has implemented the Baby Friendly Hospital Initiative (BFHI), developed by the WHO and United Nations Children’s Fund (UNICEF) in 1990 in order to address poor breastfeeding practices in maternity wards [8, 9]. This initiative emphasizes implementation at the hospital level and promotion breastfeeding in hospitals. However, the impact of BFHI is limited in less developed countries like Kenya, where more than half the women, especially the poor still deliver at home [10, 11]. Therefore, to extend the benefits of BFHI to all mothers in the community, Kenya has explored adoption of the Baby Friendly Community Initiative (BFCI) [12].

BFCI is a community-based initiative that was developed to expand on the 10th step of BFHI which focuses on supporting breastfeeding mothers after they have left the health facility. The Government of Kenya through the Ministry of Health is considering the adoption of BFCI which entails eight steps of BFCI in achieving the goal of supporting breastfeeding mothers when they go back to their homes [13]. The eight steps are as presented in Table 1 below.

**Table 1:** Eight Steps of Baby-Friendly Community Initiative

1. Have a written Maternal Infant and Young Child Nutrition (MIYCN) policy summary statement consistently communicated to health care providers, community health workers and community members; 2. Train health care providers and community health volunteers on MIYCN policy, in order to equip them with necessary skills to implement the policy; 3. Promote and support optimal maternal nutrition among women and their families; 4. Educate all pregnant women and lactating women and their families on benefits of breastfeeding to all parties and risks associated with artificial feeding; 5. Help mothers to initiate breastfeeding their children soon after delivery; within 1 h of birth and support them to maintain exclusive breastfeeding for 6 months. Address any problems related to breastfeeding; 6. Encourage mothers to continue breastfeeding their children up to 2 years or beyond, in conjunction with the appropriate, adequate and safe complementary feeding while providing holistic care and stimulation of the child; 7. Provide a friendly environment, supportive of breastfeeding families; 8. Promote collaboration among healthcare staff, community mother-support groups, mother-to-mother support groups and the members of the local community.	

Adapted from Ministry of Health, Kenya [13]

The overall aim of BFCI is to promote breastfeeding, complementary feeding, maternal nutrition using locally available foods and improve sanitation and hygiene [14]. The 6th step of BFCI centers on ensuring that breastfeeding beyond 6 months until 2 years or more is sustained and that timely, appropriate, adequate and safe complementary feeding is maintained [12]. Activities such as the establishment of mother to mother support groups, cooking demonstrations, home visitations, inclusion of spouses and grandmothers in support groups and the introduction of income generating projects such as kitchen gardens are all components of this initiative [15, 16]. Knowledge of appropriate exclusive and continued breastfeeding duration, amount of food to feed a child based on their age, recommended food groups and feeding during illness have been used as indicators to develop messages for counseling mothers. Evaluation of the effectiveness of BFCI on complementary feeding is a very important aspect that has not been well addressed. This study therefore aimed at assessing complementary feeding practices after BFCI implementation in Koibatek, a rural setting in Kenya.

## Methods

### Study design

This study was part of a large cluster randomized controlled trial that included a total of 812 mother-infant pairs, half of whom were in the intervention group [12]. This sample size was derived by considering the cluster randomized design [17]. By using a precision level of 5% (two sided t-test), a power of 80% and adjusting for expected design effect (3.15) based on correlation coefficient of 0.035 and a cluster size of 62.5, a sample size of 738 was derived. Adding 10% to cater for loss to follow up, it was estimated that a sample size of 812 would be sufficient to determine the effect of the intervention on infant feeding practices in the community.

Evaluation of the trial was carried out in a subset of the cohort at 20 months.

The intervention group received the BFCI intervention which was composed of a) home visitations to offer personalized counseling and support on infant feeding; b) establishment of mother support groups; c) provision of infant feeding education materials and d) income generating activities, while the control group received standard care which comprised of usual services offered in health facilities such as standard counseling on immunization, general nutrition, ante-natal, post-delivery and hygiene. Nutrition education/counseling conducted in the intervention group covered; immediate skin to skin contact of a mother and her baby after birth, initiation of breastfeeding, exclusive breastfeeding, mothers’ nutrition, introduction of complementary feeding, appropriate feeding frequency, quality and quantity, as well as hygiene and responsive feeding which ensures interaction and bonding between mother and child during feeding.

The BFCI project comprised 13 community units: six in the intervention arm (Solian, Simotwet, Esageri, Kiptuno, Toniok and Tugumoi) and seven in the control group (Timboroa, Makutano, Poror, Shauri, Arama, Torongo and AIC). Each of these community units was linked to a health facility.

### Study site

The study was conducted in six community units in Koibatek Sub-County, in Baringo County. Three of the community units were randomly chosen from the six that comprised the intervention units, while the other three units were picked from the control arm.

Koibatek County is approximately 222 km from Nairobi city and 63 km from Nakuru town.

### Study population

The target population were mothers with children aged 6–23 months attending health facilities that served the community units involved in the BFCI trial in Koibatek Sub-County, Baringo County.

### Sample size

A total of 294 mother and child pairs were determined as sufficient sample size to estimate the effect of the BFCI on complementary feeding among children aged 6–23 months. The sample size was calculated using the formula proposed by Yamane [18]. Sample size calculation was based on the 812 mother and child pairs included in the main study. We used a level of precison of 0.05, a 95% confidence level and 812 mother-infant pairs as the size of population. This yielded a sample size of 267. An additional 10% of the 267 mother and child pairs were added to cater for non-response and natural attrition [19]. Therefore, the final sample was estimated to be 294 mother-child pairs.

### Sampling technique

**Fig. 1:**
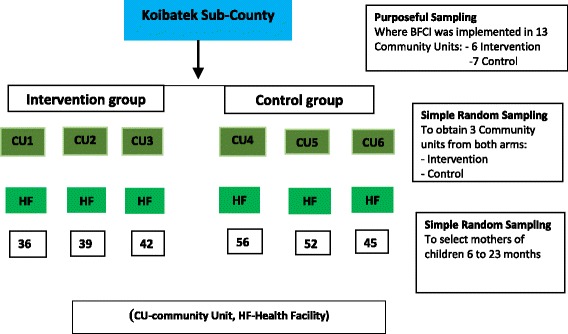
Study sample selection process

As shown in Fig. 1, the African Population and Health Research Center (APHRC) and Kenyatta University in collaboration with the Sub-County of Koibatek implemented the BFCI pilot study in Koibatek Sub-county [12]. This was the first such program in Kenya. A two-stage sampling procedure was used in the selection of the study participants. First, simple random sampling (balloting) was used to select three community units each from the intervention and control arms. The intervention arm had six community units: Solian, Kiptuno, Simotwet, Toniok, Esageri, and Tugumoi while the control group had seven community units: Timboroa, Makutano, Poror, Shauri, Arama, Torongo and Aic. The six community units included in the study were: Intervention; Solian, Simotwet and Toniok and Control; Timboroa, Aic and Shauri.

In the second stage, the sample size of 294 was distributed proportionately to size of respondents that were recruited in the main BFCI study. However, the study obtained 270 mother-infant pairs, which was a response rate of 92%. The number obtained from each community unit is as shown in the last column in Table 2.

**Table 2:** Distribution of study respondents participating in the BFCI in Koibatek, Kenya

Community unit	Population in BFCI	Proposed to be selected	Actual participation in study
Solian	65	43	36
Simotwet	63	42	39
Toniok	63	42	42
Timboroa	100	66	56
Aic	83	55	52
Shauri	70	46	45
TOTAL	444	294	270

The 270 mothers-infant pairs who attend the affiliated health facilities were selected from their six respective communities.

The study sample selection process is presented in Fig. 1.

### Inclusion and exclusion criteria

Mothers with children 6–23 months who had been recruited in the BFCI project, who were found in their respective community units where they had been initially recruited were included in the study. Mothers who moved/crossed over from the one treatment arm to another that is, from control to intervention and vice versa and those who had benefitted from the intervention but whose children had died before we reached them were excluded from the study.

### Research instrument

Data was collected using a structured questionnaire which was administered by research assistants. The questionnaire was adapted from the Kenya Ministry of Health’s Maternal, Infant and Young Child Nutrition questionnaire. The questionnaire was previously validated during its use in nationwide infant feeding surveys in Kenya. The questionnaire captured among other things demographic characteristics of the study participants (age, religion, level of education, marital status, main household income source) and the complementary feeding practices.

### Measurement variables

#### Complementary feeding practices

Complementary feeding practices were assessed based on WHO core infant and young child feeding indicators: minimum meal frequency, minimum dietary diversity and minimum acceptable diet following a 24 h dietary recall [21].

##### Minimum meal frequency (MMF)

Minimum meal frequency was based on age of child, and whether the child was still breastfeeding and the number of times it was fed solid and semi-solid foods (thick consistency foods). Minimum meal frequency was defined as: Breastfed infants aged 6–8 months old eating two times a day, breastfed children aged 9–23 months eating three times per day and four times daily for children aged 6–23 months who are not breastfed [20].

##### Minimum dietary diversity (MDD)

According to FAO 2014 guidelines, feeding of children 6–23 months should focus on including foods from the seven food groups: Cereals/grains, roots, tubers and plantains; legumes, nuts, seeds; dairy products; meat; eggs; Fruits and vegetables rich in Vitamin A; other fruits and vegetables. FAO recommends that a breastfed child aged between 6 and 23 months should consume from a minimum of four food groups. This criteria was used to define minimum dietary diversity.

##### Minimum acceptable diet (MAD)

Minimum acceptable diet was derived from both the minimum meal frequency and minimum dietary diversity. WHO defines MAD as all children 6–23 months who have achieved minimum meal frequency and have attained minimum dietary diversity [21].

### Data analysis

Data was entered in Statistical Package for Social Sciences (SPSS) version 17.0 for cleaning and coding. The analysis was performed using Stata version 14 (Stata Corporation, College Station, TX). Descriptive statistics were presented in the form of tables and graphs. Pearson’s chi-squared test was used to assess the differences between groups based on their socio-demographic characteristics (which include mother’s age, education level, marital status, religion and main source of income) and trial arm (control or intervention) and complementary feeding practices (MMF, MDD, MAD). *P*-value of < 0.05 was considered significant.

Logistic regression was used to investigate the association between the socio-demographic characteristics and trial arm (control or intervention) and complementary feeding practices (attained MMF, MDD, MAD). The model controlled for variables including mother’s age, education level, marital status, religion and main source of income that might also influence the outcome (complementary feeding practices). The strength of association between the factors and outcome variable, in terms of odds ratio together with the 95% confidence intervals are presented as appropriate.

## Results

Two-hundred and seventy mother-infant pairs were included in the study, with a response rate of 92%.

### Characteristics of the study population

Mothers with children in the age category of 6–12 months made up 53% of the study population while 47% of the population were mothers with children between 13 and 23 months. Male and female children were almost equally represented (54% females).

Most mothers who participated in the study were aged between 21 and 30 years (62%) while significantly fewer mothers were aged 41–49 years (4%). Most mothers were married (94%) and Christians (82%). The highest education level attained by most mothers was secondary level/high school (48%), 22 (8%) number of women had obtained a college diploma only one (0.4%) had attained a university degree. There were more mothers in the control group (47/117) who were Muslims compared to those in intervention group (3/153). One of the community units that made the control unit was predominantly inhabited by the Nubian community, who practice Islam. More about study participants is shown in Table 3.

**Table 3:** Child and maternal socio-demographic characteristics of study participants (Intervention = 117 and Control = 153)

	Intervention	Control	Overall (*N* = 270)
Child characteristics			
Gender of child			
Male	53	71	124 (45.9%)
Female	64	82	146 (54.1%)
Child’s age category			
6–12 months	50	92	142 (52.6%)
13–23 months	67	61	128 (47.4%)
Maternal characteristics			
Age of mother			
<20	11	29	40 (14.8%)
21–30	86	82	168 (62.2%)
31–40	13	37	50 (18.5%)
41–49	07	5	12 (4.4%)
Marital status			
Married	107	147	254 (94.1%)
Single	7	6	13 (4.8%)
Separated	3	0	3 (1.1%)
Religion			
Christian	114	106	220 (81.5%)
Muslim	3	47	50 (18.5%)

### Complementary feeding practices

#### Minimum meal frequency

**Fig. 2:**
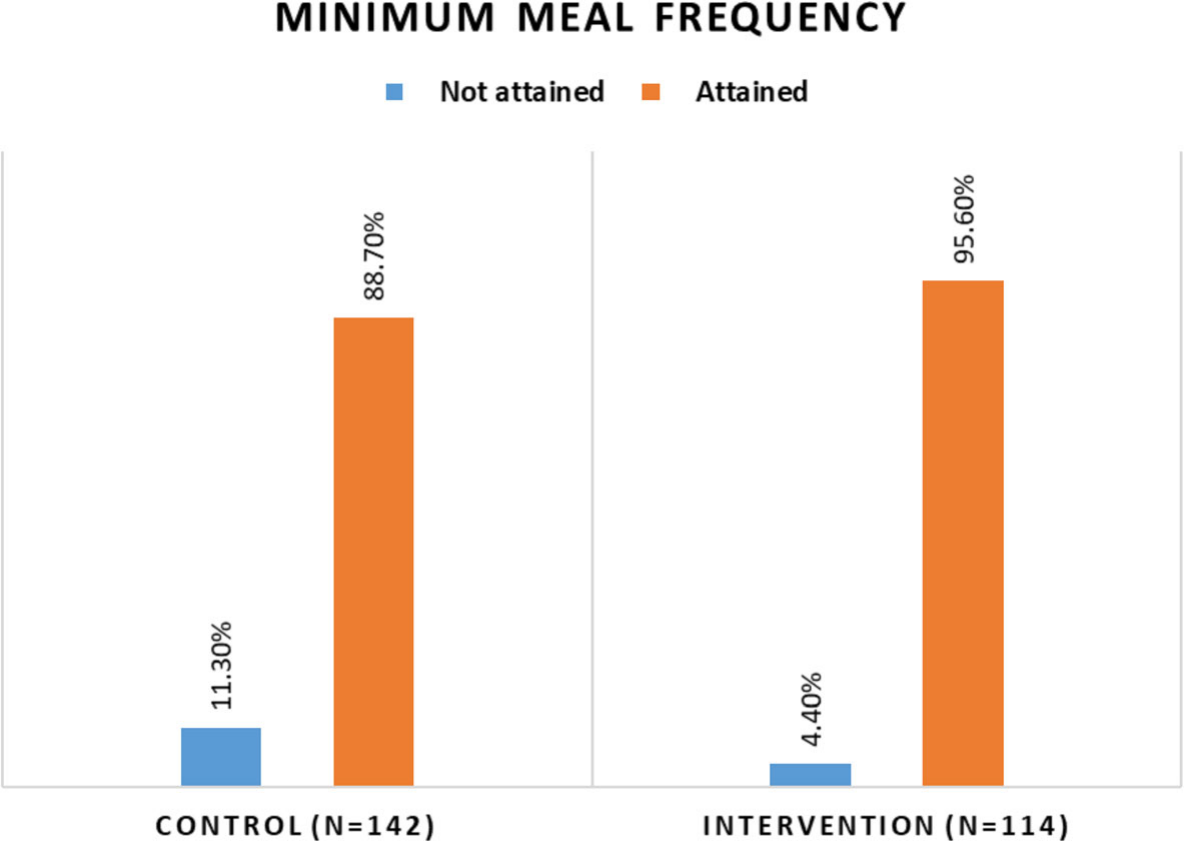
Comparison of minimum meal frequency attained by children 6–23 months between intervention and control groups Chi-square test; *p* = 0.05 at 95% Cl; *p* = 0.046

More children in the intervention group (96%) achieved the minimum meal frequency compared to 89% in the control group (*p* = 0.046) (Fig. 2).

#### Minimum dietary diversity

**Fig. 3:**
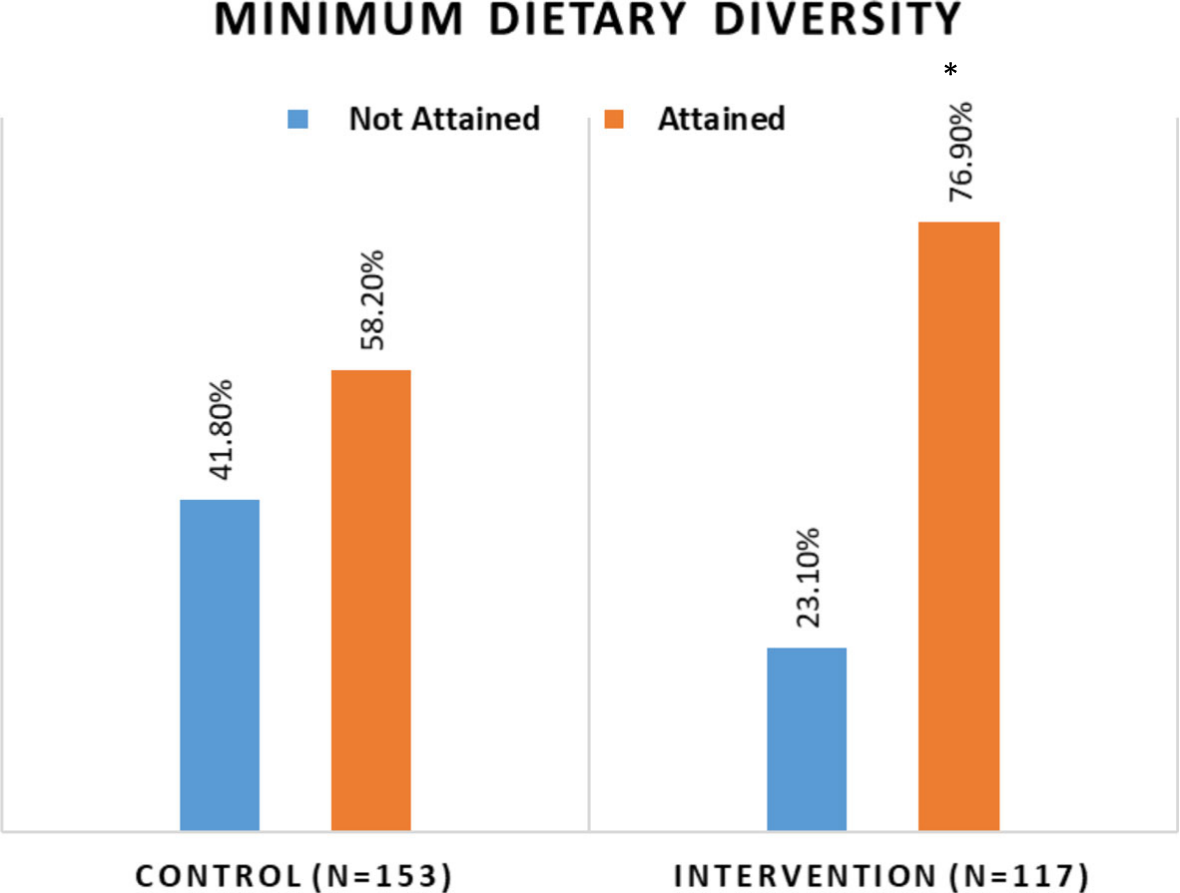
Comparison of minimum dietary diversity attained by children 6–23 months between intervention and control groups. Chi-square test: *p* = 0.05 at 95% Cl; * *p* = 0.001

Seventy seven percent of children in the intervention group (77%) had attained the minimum dietary diversity compared to 58% in the control group (*p* = 0.001) (Fig. 3).

In general, more children from the intervention group than controls consumed food from at least four food groups. Overall, 67 and 23% of the children in the study consumed Vitamin A rich and Iron-rich foods respectively. Seventy-eight percent in the intervention arm consumed vitamin A rich fruits and vegetables compared to 56% in the control group (*p* = 0.001). More children in the intervention group consumed legumes/nuts, meat and dairy product food groups compared to controls and the differences were statistically significant (*p* = 0.003, 0.015, 0.005 respectively). There was no significant difference in consumption of grains/cereals, roots and tubers. More children in the intervention group (30%) consumed Iron-rich foods compared to those in control group (17%) (*p* = 0.015).

#### Minimum acceptable diet

**Fig. 4:**
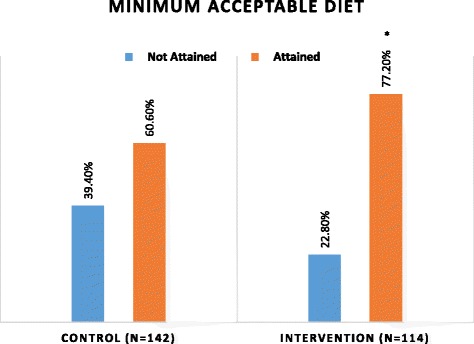
Comparison of minimum acceptable diet attained by children 6–23 months between intervention and control groups. Chi-square test; *p* = 0.05 at 95% Cl; * *p* = 0.005

A bigger proportion of children in the intervention group (77%) attained the minimum acceptable diet compared to the control group (61%) (*p* = 0.005) (Fig. 4).

#### Relationship between BFCI and complementary feeding practices

##### Minimum meal frequency

The odds of attaining minimum meal frequency were 15 times higher for the intervention group compared to the control (OR: 14.84; 95%CI 2.75–79.9), when controlling for mother’s age, education level, marital status, religion and main source of income (*p* = 0.002) (Table 4).

**Table 4:** Relationship between BFCI intervention and minimum meal frequency

Variable	Unadjusted OR (95% Cl)	*p* value	Adjusted OR (95% Cl)	*p* value
Study group				
Control	1.00		1.00	
Intervention	2.81 (0.99–7.93)	0.051	14.84 (2.75–79.90)	0.002
Age of mother				
<20 years	1.00		1.00	
21–30 years	0.55 (0.12–2.57)	0.456	1.35 (0.10–17.88)	0.818
31–40 years	0.18 (0.06–0.42)	0.100	0.15 (0.23–0.78)	0.056
41–49 years	0.77 (0.01–0.48)	0.006	0.01 (0.00–0.13)	0.000
Education level				
No education	1.00		1.00	
Primary	0.93 (0.36–2.39)	0.888	0.17 (0.04–0.17)	0.015
Secondary/Tertiary	0.90 (0.18–4.51)	0.907	0.06 (0.01–0.76)	0.029
Marital status				
Unmarried	1.00		1.00	
Married	1.00 (1.12–8.22)	0.993	2.35 (0.01–44.8)	0.569
Religion				
Christian	1.00		1.00	
Muslim	0.70 (0.24–2.02)	0.515	2.86 (0.70–11.68)	0.144
Income source				
No income	1.00		1.00	
Merchant/trader	1.21 (0.82–12.77)	0.054	1.01 (0.62–2.25)	0.675
Permanent job	1.37 (0.38–4.90)		4.64 (0.47–35.70)	0.185

Logistic regression analysis; *p* = 0.05 at 95% CI

The multivariate model was adjusted for mother’s age, education level, marital status, religion and main source of income

##### Minimum dietary diversity

The odds of attaining minimum dietary diversity were five times greater in the intervention group compared to the control group (OR: 4.95; 95% CI 2.44–10.03), when mother’s age, education level, marital status, religion and main source of income were controlled for (*p* = < 0.001) (Table 5).

**Table 5:** Relationship between BFCI intervention minimum dietary diversity

Variable	Unadjusted OR (95% Cl)	*p* value	Adjusted OR (95% Cl)	*p* value
Study group				
Control	1.00		1.00	
Intervention	2.76 (1.63–4.68)	< 0.001	4.95 (2.44–10.03)	< 0.001
Age of mother				
<20 years	1.00		1.00	
21–30 years	0.39 (0.16–0.94)	0.036	0.26 (0.60–1.17)	0.080
31–40 years	0.16 (0.06–0.44)	0.000	0.13 (0.27–0.68)	0.056
41–49 years	0.10 (0.02–0.45)	0.002	0.03 (0.01–0.15)	0.984
Education level				
No education	1.00		1.00	
Primary	1.32 (0.79–2.19)	0.286	0.51 (0.26–0.97)	0.141
Secondary/Tertiary	5.15 (1.45–18.29)	0.167	1.42 (0.34–5.95)	0.628
Marital status				
Unmarried	1.00		1.00	
Married	1.70 (0.61–4.68)	0.303	1.91 (0.53–6.85)	0.320
Religion				
Christian	1.00		1.00	
Muslim	0.98 (0.52–1.86)	0.971	3.78 (1.69–8.49)	0.001
Income source				
No income	1.00		1.00	
Merchant/trader	4.21 (1.20–14.76)	0.024	3.35 (0.91–3.56)	0.985
Permanent job	1.45 (0.72–2.91)	0.291	1.37 (0.10–1.41)	0.313

Logistic regression analysis; *p* = 0.05 at 95% CI

The multivariate model was adjusted for mother’s age, education level, marital status, religion and main source of income

##### Minimum acceptable diet

The odds of attaining a minimum acceptable diet were almost five times higher for the intervention group compared to the control group (OR: 4.61; 95%CI 2.17–9.78), when mother’s age, education level, marital status, religion and main source of income were adjusted (*p* = < 0.001) (Table 6).

**Table 6:** Relationship between BFCI and minimum acceptable diet

Variable	Unadjusted OR (95% Cl)	*p* value	Adjusted OR (95% Cl)	*p* value
Study group				
Control	1.00		1.00	
Intervention	2.66 (1.55–4.57)	< 0.001	4.61 (2.17–9.78)	< 0.001
Age of mother				
<20 years	1.00		1.00	
21–30 years	0.46 (0.18–1.11)	0.086	0.21 (0.49–0.91)	0.037
31–40 years	0.18 (0.06–0.49)	0.001	0.11 (0.01–0.67)	0.057
41–49 years	0.11 (0.02–0.48)	0.003	0.03 (0.01–0.17)	0.987
Education level				
No education	1.00		1.00	
Primary	1.28 (0.75–2.19)	0.349	0.51 (0.25–1.00)	0.051
Secondary/Tertiary	3.38 (1.08–10.58)	0.036	1.01 (0.26–3.87)	0.986
Marital status				
Unmarried	1.00		1.00	
Married	1.25 (0.38–4.05)	0.710	1.01 (0.23–4.33)	0.346
Religion				
Christian	1.00		1.00	
Muslim	0.81 (0.42–1.46)	0.548	2.87 (1.24–6.62)	0.013
Income source				
No income	1.00		1.00	
Merchant/trader	3.41 (0.96–12.13)	0.057	1.64 (0.52–1.99)	0.989
Permanent job	1.19 (0.59–2.38)	0.611	0.49 (0.14–1.61)	0.242

Logistic regression analysis; *p* = 0.05 at 95% CI

The multivariate model was adjusted for mother’s age, education level, marital status, religion and main source of income

## Discussion

The findings of this study demonstrate that the Baby Friendly Community Initiative had a positive impact on complementary feeding practices. Mothers in the intervention groups had significantly better frequency of feeding, dietary diversity and minimum acceptable diet compared to control groups. These results remained significant even after adjusting for socio-demographic factors; the age of mother, marital status, education level, religion and the main source of income. These findings are supported by other studies on community-based interventions geared towards improved child health outcomes.

Possible explanations for these results include the wide acceptance and support for the BCFI program among the community, spouses and relatives. The outcomes of this study were similar to those reported in an intervention in Western Kenya that aimed at providing social support to the mother by enlisting fathers and grandmothers in encouraging specific practices for infant feeding. Significant improvements were reported for minimum meal frequency (OR 1.14, 95% CI:1.00–1.30, *p* = 0.047) and minimum dietary diversity (OR 1.19, 95%CI:1.01–1.40, *p* = 0.04) [22].

A second explanation proposed for the success of the BFCI is the fact that it was developed as a community-based nutrition education program. Behavior change through nutrition education has been successful in many localities. A cluster randomized trial conducted in rural China found that following a community-based nutrition education program, there was an improvement in dietary diversity and meal frequency in the intervention group [23]. Similarly, a cluster randomized trial in India found that community-based educational interventions improved dietary intakes and growth in children under 2 years [24]. An integrated community nutrition program in rural Vietnam that employed a community mobilization strategy found that the intervention positively impacted infant feeding practices such as minimum meal frequency [25].

A third element that may help explain the success of BFCI is the central role of mother and peer support groups. This approach is a major component of the BFCI for improving feeding practices of infants and children [13]. Overall, findings of this study indicate that a high proportion of children in the intervention group, whose mothers were members of mother support groups were fed better in comparison to children in the control group. A prospective study in Lalitpur District, India that included 1426 mother-infant pairs evaluated the impact of mother support groups as part of BFCI intervention to promote optimal infant feeding practices. The study found that the intervention had a significant impact on the improvement of infant feeding practices. For instance, there was a significant improvement in the age-appropriate initiation of complementary feeding, 5 years after the intervention (96%) compared to 54% in pre-intervention period (OR 22.9, 95%CI:11.8–44.1, *p* = < 0.001) [26]. However, a cluster-randomized controlled study conducted in Bangladesh that assessed the influence of a participatory womens’ group on infant feeding and other health outcomes in children did not find any differences in dietary diversity scores [27].

Another important component of BFCI is nutrition education for health and nutrition care workers and community health workers. A community randomized trial in rural Haryana, India that included an intervention group where health and nutrition workers in the community were trained on counseling on appropriate infant feeding, such as appropriate complementary feeding, in addition to community and health workers mobilization, found that intervention group had better complementary feeding practices (45%) when compared to control group (11.5%) [28].

This study found that a statistically significantly higher number of children in the intervention group compared to control consumed vitamin A rich fruits and vegetables, legumes and nuts, meat and dairy products. Similarly, a randomized control trial in the disadvantaged city of London boroughs that included 212 mother-infant pairs found that following a social support intervention that comprised of home visits by trained health workers, a significantly higher proportion of children in the intervention group consumed more fruits and vegetables compared to the children in control [29].

Findings of this study are further supported by a systematic review that reviewed 15 intervention studies, and resolved that education interventions considering culture and local resources can successfully improve complementary feeding practices [30].

In this study, there were more Muslim mothers in the control group compared to the intervention group. It was put into consideration that mothers in control group could have better breastfeeding practices when compared to those in intervention group, since Muslim religion directs that mothers breastfeed their children. However, this study found that complementary feeding practices are better in the intervention group compared to the control group. It is possible that Islam instructs breastfeeding but may not be clear on appropriate complementary feeding.

## Conclusion

The findings of this study reveal that BFCI intervention in Koibatek, Kenya has been successful in improving complementary feeding practices. As manifested in this study, scaling up BFCI intervention would improve child feeding, especially dietary diversity and meal frequencies thus ensuring nutritional adequacy. Moreover, strengthening and prioritizing BFCI intervention throughout the country by the Ministry of Health could have a significant impact on overall child health outcomes in Kenya. Use of the BFCI has repeatedly been associated with improved breastfeeding and complementary feeding practices. The Ministry of Health should explore adoption of mother support groups, community mother support groups and home visits by trained community health workers as ways of promoting optimal health and nutrition of children. Likewise, stakeholders should invest in interventions geared towards improving maternal knowledge, which will consequently improve infant feeding, nutrition and health status. These interventions include designing and funding of nutrition education programs for health facility workers and community health workers who are often in contact with mothers.

Limitations of this study include self-report and recall bias as the study relied on mothers’ memory and therefore the information given could not be verified to be correct. Due to the geographical location where the study was carried out, a rural setting, the findings may not be generalizable for non-rural settings. There have been previous Government interventions geared towards addressing child and maternal health issues in both intervention and control sites, therefore changes observed cannot only be attributed to the effects of BFCI.

The strengths of this study include: its rigorous scientific design using a randomized control intervention that included a large sample size of 270 mother-child pairs. Other stengths include the fact that the BFCI is a community-based intervention, so it can easily be integrated into existing community health systems thus increasing chances for sustainability. To the best of our knowledge, this study is the first in Kenya that evaluated the impact of a BFCI intervention on complementary feeding of children aged 6–23 months. The results of this study make a meaningful contribution to the existing knowledge regarding community intervention programs and should encourage future research and be used to inform policy.

In order to strengthen these findings, it is crucial that additional evaluations be carried out in randomized control trials in other sites in Kenya that are implementing the BFCI. Positive outcomes on infant feeding and other child health indicators would provide further evidence-based information to support the BFCI interventions and allow results to be generalizable to other settings. In future studies on BFCI, it would be prudent to implement the eight steps in such a way that would allow researchers to assess which step was more useful.

## Abbreviations

BFCI: Baby Friendly Community Initiative
BFHI: Baby Friendly Hospital Initiative
CU: Community Unit
FAO: Food & Agriculture Organization of the United Nations
HF: Health Facility
KDHS: Kenya Demographic Health Survey
KNBS: Kenya National Bureau of Statistics
MAD: Minimum Acceptable Diet
MCHIP: Maternal and Child Health Integrated Program
MDD: Minimum Dietary Diversity
MMF: Minimum Meal Frequency
MOH: Ministry of Health
NACOSTI: National Commission for Science, Technology and Innovation
SPSS: Statistical Package for Social Sciences
UNICEF: United Nations Children’s Fund
USAID: United States Agency for International Development
WHO: World Health Organization
